# Cytosolic and mitochondrial ribosomal proteins mediate the locust phase transition via divergence of translational profiles

**DOI:** 10.1073/pnas.2216851120

**Published:** 2023-01-26

**Authors:** Jing Li, Liya Wei, Yongsheng Wang, Haikang Zhang, Pengcheng Yang, Zhangwu Zhao, Le Kang

**Affiliations:** ^a^College of Life Science, Institute of Life Science and Green Development, Hebei University, Baoding 071002, China; ^b^Beijing Institutes of Life Science, Chinese Academy of Sciences, Beijing 100101, China; ^c^Institute of Zoology, State Key Laboratory of Integrated Management of Pest Insects and Rodents, Chinese Academy of Sciences, Beijing 100101, China

**Keywords:** translational regulation, cytosolic and mitochondrial ribosomal proteins, phase transition, migratory locust, behavioral aggregation

## Abstract

The phase transition from solitary to gregarious locusts is crucial in outbreaks of locust plague, which threaten agricultural yield and food security. Research on the regulatory mechanisms of phase transition in locusts has focused primarily on the transcriptional or posttranslational level. However, the translational regulation of phase transition is unexplored. Here, we show a phase-dependent pattern at the translation level, which exhibits different polysome profiles between gregarious and solitary locusts. The gregarious locusts exhibit significant increases in 60S and polyribosomes, while solitary locusts possess higher peaks of the monoribosome and a specific “halfmer.” The polysome profiles, a molecular phenotype, respond to changes in population density. In gregarious locusts, ten genes involved in the cytosolic ribosome pathway exhibited increased translational efficiency (TE). In solitary locusts, five genes from the mitochondrial ribosome pathway displayed increased TE. The high expression of large ribosomal protein 7 at the translational level promotes accumulation of the free 60S ribosomal subunit in gregarious locusts, while solitary locusts employ mitochondrial small ribosomal protein 18c to induce the assembly of mitochondrial ribosomes, causing divergence of the translational profiles and behavioral transition. This study reveals the translational regulatory mechanism of locust phase transition, in which the locusts employ divergent ribosome pathways to cope with changes in population density.

## Significance

Outbreaks of locust plagues are largely attributed to the phase transition from solitary to gregarious locusts. Many studies have demonstrated transcriptional and posttranslational regulation in phase change. However, the translational regulation in the locust phase transition is unclear. Here, we found plasticity in polysome profiles between solitary and gregarious locusts. A divergence with ribosomal proteins from cytoplasm and mitochondria modulates behavioral features of gregarious and solitary locusts. The findings reveal that the population density of locusts, as an environmental signal, can initiate translational regulation for the phenotypic plasticity of insects. Important clues in searching for targets to control pests are also provided. Insights into energy metabolism regulation at the translational level in eukaryotes are presented as well.

Locusts exhibit striking phenotypic plasticity in response to changes in population density. The devastating gregarious swarms are formed by a high population density of locusts. Severe outbreaks of locust infestations are attributed to phase changes from low-density solitary to high-density gregarious locusts. Phase-related morphological, physiological, ethological, and neurobiological phenotypes have been highlighted (1, 2). Previous studies have shown that the locust phase transition involves a complex regulatory network of multiple factors and levels affecting behavior, flight, reproduction, immunity, body color, and defense (1, 3–7).

Behavioral transition is the fastest and most remarkable response to changes in population density in locusts, and it plays a key role in the formation of large-scale locust swarms (1, 8). Several critical coding and noncoding genes in the dopamine pathway have been identified at the transcriptional, posttranscriptional, or posttranslational levels as being involved in the modulation of behavioral phase changes in the migratory locust (9–13). Notably, the dopamine synthesis genes *henna* and *pale* (11) are both negatively regulated by miR-133 at the posttranscriptional level, but miR-133 only mediates the translational repression of *pale* by binding to its 3′-*untranslated region* (UTR), which leads to the inhibition of behavioral aggregation (14). In the migratory locust, the major differentially expressed transcripts between solitary and gregarious locusts include ribosomal proteins (RPs) and translation initiation factors for translational regulation (15). Similarly, three RPs and translation initiation factor 3 were found to be differentially expressed in proteome data of gregarious and solitary locusts (16). Importantly, the high translational efficiency (TE) of *brm* targeted by miR-276 promotes egg-hatching synchrony in gregarious locusts (17). These data imply that the locust phase transition is closely related to translational regulation. However, the molecular mechanism of translational regulation is still unclear.

A slight correlation exists between mRNA and protein abundance (18, 19), in which the protein abundance is better predicted by the rate of protein synthesis at the ribosome (20). Thus, translational regulation predominantly controls protein production. Currently, the nucleotide resolution approach of in-depth translational analysis in vivo can monitor genome-wide protein translation and gene-specific ribosome density profiles (21). Subsequently, translation profiles of the genome-wide life cycle of fruit flies have been investigated by the ribosome profiling sequencing (Ribo-seq) strategy (22–24). Global protein homeostasis by translational regulation not only plays a crucial role in cell growth, differentiation, and organismal development but also can be quickly adjusted as living organisms respond to environmental signal changes (25). Dynamic translational control is documented in plant adaptation to environmental signal changes, including light, hypoxia, drought, ethylene, heat stress, nitrogen starvation, and pathogen challenge (26–28). Thus, decoding the population density of locusts as an environmental signal that mediates the translational regulation of locust behavior is important in searching for targets to control locust plagues.

In this study, we first established dynamic ribosome profiling of the locust phase transition. Then, mRNA-seq and Ribo-seq were used to evaluate the TE of the given genes. We found that the difference in polysome profiles was due to the functional divergence of the cytosolic and mitochondrial ribosome pathways between solitary and gregarious locusts. We verified that large ribosomal protein 7 (RPL7) and mitochondrial small ribosomal protein 18c (MRPS18c), by translational regulation, affected the ribosome profiles and behavioral shift between solitary and gregarious locusts. Our data provide some insight into the phenotypic plasticity of locusts at translational level and potential targets for pesticide development.

## Results

### Differentially Expressed Ribosome-Related Genes between Solitary and Gregarious Locusts.

We reanalyzed the locust transcriptome data of the whole body, different tissues, and developmental stages as well as solitarization and gregarization density treatments to examine the possible involvement of translational regulation in the phase changes in locusts (15, 29). The results showed that the ribosome pathway contained the most differentially expressed genes and that the up-regulated pathways were significantly enriched in ribosomes and translation pathways in gregarious locusts compared with solitary locusts (*SI Appendix*, Tables S1 and S2 and Fig. S1*A*). In particular, the variable density treatments of solitarization and gregarization displayed functional enrichment of ribosomes (*SI Appendix*, Fig. S1*B*). Therefore, differentially expressed ribosome-related genes between gregarious and solitary locusts imply a possible regulatory effect in locust phase transition.

### Differences in Polysome Profiles between Gregarious and Solitary Locusts.

**Fig. 1.**
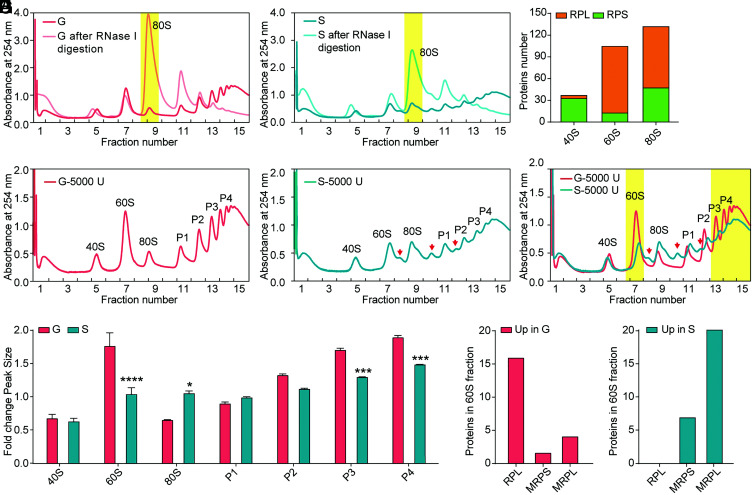
Polysome profile differences between gregarious and solitary locusts. (*A*) Absorbance (A254 nm) of sucrose density gradient-fractionated ribosomes from RNase I- or mock-treated (control) gregarious locusts. (*B*) Absorbance (A254 nm) of sucrose density gradient fractionated ribosomes from RNase I- or mock-treated (control) solitary locusts. (*C*) Mass spectrometry (MS/MS) analysis of proteins in fractions 5, 7, and 9 of the sucrose density gradient. (*D*) Absorbance (A254 nm) of sucrose density gradient fractions measured from ribosomes in gregarious locusts. (*E*) Absorbance (A254 nm) of sucrose density gradient fractions measured from ribosomes in solitary locusts. (*F*) Different polysome profiles from gregarious and solitary locusts. The X-axis indicates thetop (fraction 1) to the bottom (fraction 15)  from 0 mm to 75 mm of the 5 to 50% sucrose gradient. The Y-axis indicates the absorbance (A254 nm) from ribosomes. The red arrowheads indicate specific peaks in solitary locusts. Yellow regions highlight 60S ribosomal subunits and polyribosomes. (*G*) Quantification of polysome peak sizes of gregarious and solitary locusts in representative experiments with n = 3, normalized to the P1 peak of solitary locusts. The normalized method was performed as in a previous study (30). Bars represent the mean ± SEM, and significance was tested with Student’s *t* test, with *P < 0.05, **P < 0.01, ***P < 0.001, ****P < 0.0001. (*H*) The number of differential abundance proteins from 60S fractions in gregarious and solitary locusts was detected by mass spectrometry (MS/MS). The 60S fractions contain the 60S large ribosomal subunit and 60S mitochondrial ribosomes in locusts. The higher abundance of proteins (MS/MS count fold change > 1.2) in gregarious locusts than solitary locusts was defined as Up in G, while the higher abundance of proteins (MS/MS count fold change > 1.2) in solitary locusts than gregarious locusts was defined as Up in S.

We extracted ribosomes from the whole body of gregarious and solitary locusts by sucrose density gradient fractionation to monitor the differences in translational regulation associated with locust phase transition (Fig. 1 and *SI Appendix*, Fig. S2). Ribosomes were detected at 254 nm absorbance. Several steep peaks were found in gregarious locusts (Fig. 1 *A*, *D*, and *F*), whereas solitary locusts displayed relatively smooth peaks (Fig. 1 *B*, *E*, and *F*). Fractions 5, 7, and 9 from gregarious and solitary locusts were the 40S small ribosomal subunit, the 60S large ribosomal subunit, and the 80S monoribosome, respectively (Fig. 1 *A*–*C*). The profiles of 60S subunits and P3 and P4 polyribosomes peaks in gregarious locusts were significantly higher than in solitary locusts (Fig. 1 *F* and *G*). However, several special “half-mer” peaks and high monoribosome peaks appeared in the solitary locusts (Fig. 1 *F* and *G*). Furthermore, the maximum difference in 60S fraction was the large RPs in the cytoplasm up-regulated in gregarious locusts, whereas the mitochondrial RPs up-regulated in solitary locusts (Fig. 1*H*). These differences in ribosomal components between gregarious and solitary locusts suggest that translational regulation could contribute to phase divergence.

### Dynamic Polysome Profiles of Locusts Respond to Population Density Changes.

**Fig. 2.**
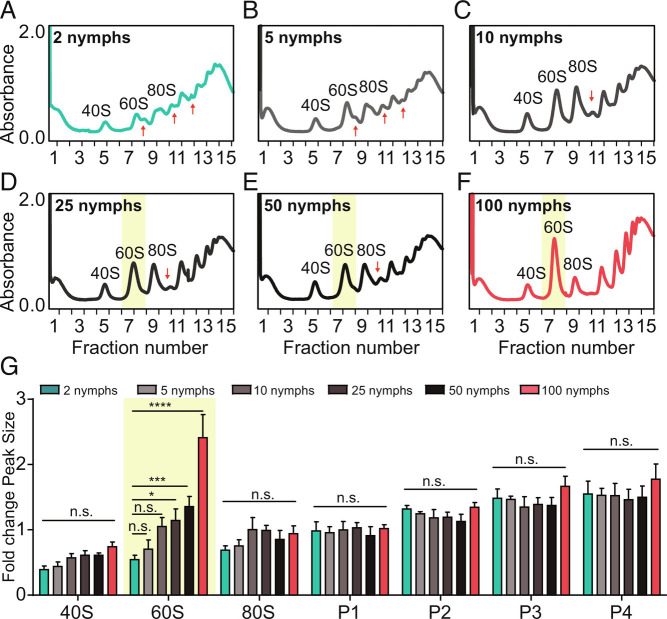
Dynamic changes in the locust polysome profile in response to population density. (*A–F*) Absorbance (A254 nm) of sucrose density gradient fractions measured from ribosomes in locusts at six different population densities, namely, 2, 5, 10, 25, 50, and 100 nymphs. The X-axis indicates the top (fraction 1) to the bottom (fraction 15)  from 0 mm to 75 mm of the 5 to 50% sucrose gradient. The Y-axis indicates the absorbance (A254 nm) of ribosomes. The red arrowheads indicate peaks specific to solitary locusts. Yellow regions highlight 60S ribosomal subunits. (*G*) Quantification of polysome peak sizes of locusts at six different population densities in representative experiments with n = 3, normalized to the P1 peak of 2 nymphs. The normalized method was performed as previously described (30). *P* values were calculated using a *t* test, with *P < 0.05, **P < 0.01, ***P < 0.001, ****P < 0.0001.

The population density determines the differentiation of locust phases. Thus, we extracted the ribosomes of the locusts under different population densities. The results showed that the polysome profiles were significantly impacted by population density (Fig. 2 *A*–*G*). The locusts exhibited typical solitary-like polysome profiles with several special half-mer peaks at the lowest population density (Fig. 2 *A*–*G* and *SI Appendix*, Fig. S3). In contrast, the locusts displayed typical gregarious-phase polysome profiles with an abundance of 60S subunits at the highest population density (Fig. 2 *F* and *G* and *SI Appendix*, Fig. S3). On the other hand, with stepwise changes from low to-high population densities, we also observed gradient declines in half-mer peaks and increases in 60S subunits (Fig. 2 *A*–*G* and *SI Appendix*, Fig. S3). Thus, these data demonstrate that the dynamic polysome profiles of locusts respond directly to population density changes.

### Ribosome Footprint Distribution in Locusts.

We evaluated the reproducibility of the sequencing data and analyzed the Ribo-seq and mRNA-seq reads in gregarious and solitary locusts to determine whether the phase transition of locusts is regulated at the translational level (*SI Appendix*, Fig. S4). The data showed a high correlation in two biological repeats and separation between gregarious and solitary samples (*SI Appendix*, Fig. S4*B*). In *SI Appendix*, Fig. S5*A*, we calculated the frequency of 26-nt, 27-nt, and 28-nt ribosomal footprints (RFs), which were above 60% as mainly RNA fragment lengths protected by ribosomes, known as RFs. Moreover, the frequency of 26-nt and 27-nt RFs were 10.6% and 38.4% in solitary locusts, and 9.4% and 36.7% in gregarious locusts, respectively. The 28-nt RFs had a frequency in gregarious locusts (17.8%) and a frequency in solitary locusts (16.8%). The 28-nt RFs showed the typical 3-nucleotide periodicity as the frequency of first position was higher than the second and third positions, while the 26-nt and 27-nt RFs also have distinct 3-nucleotide periodicity due to two and one nucleotide differences (*SI Appendix*, Fig. S5*C*). We calculated the TE as the relative ratio of Ribo-seq read RPM to mRNA-seq RPM across the same genes. Compared with solitary locusts, gregarious locusts had more genes with high TE (*SI Appendix*, Fig. S5*B*).

### Divergence of TE between Gregarious and Solitary Locusts.

**Fig. 3.**
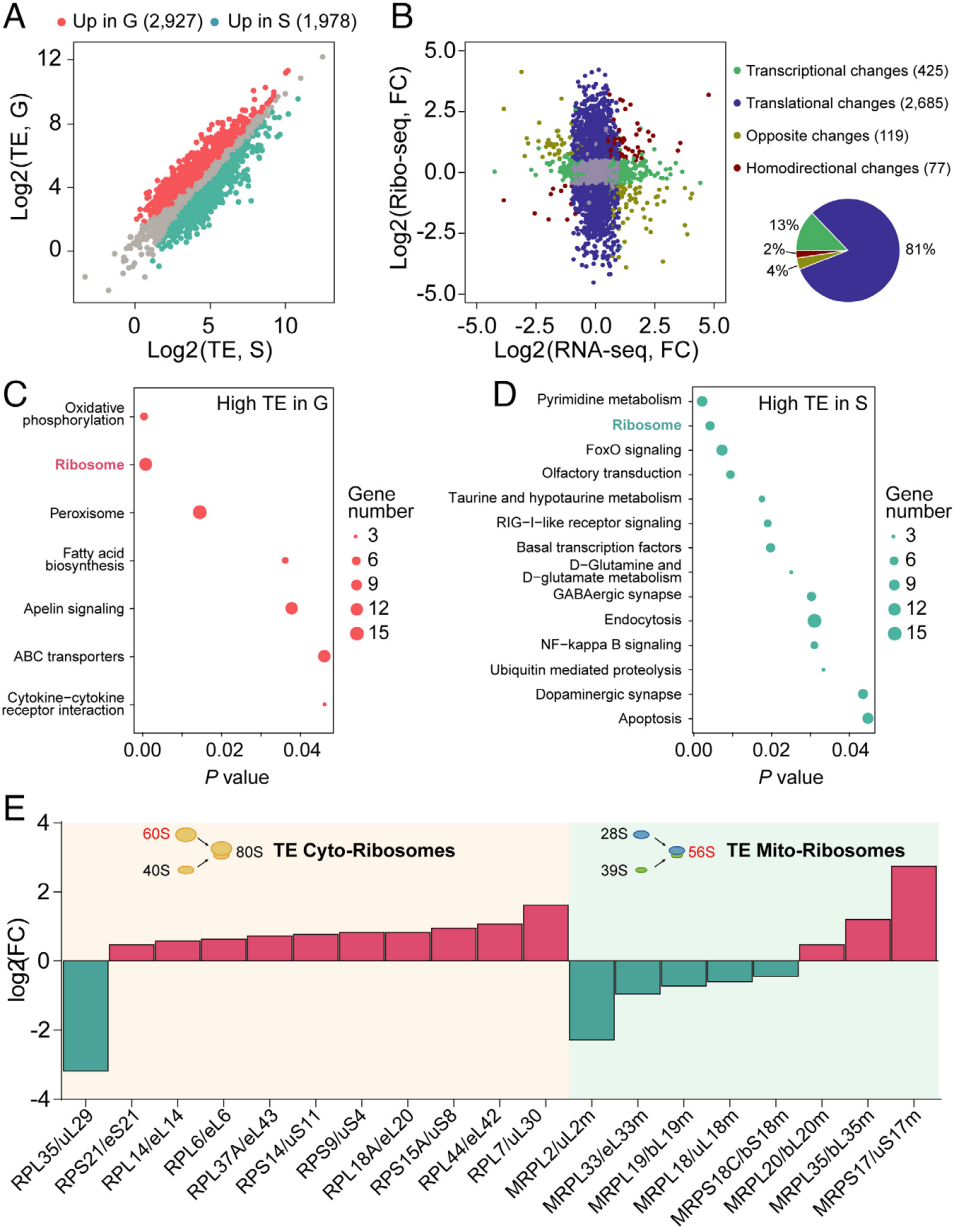
Changes in TE of divergent genes between the two phases of the migratory locusts. (*A*) Comparison of TE in gregarious and solitary locusts. Red dots represent significantly up-regulated genes in gregarious locusts (FDR < 0.01). Blue dots represent significantly up-regulated genes in solitary locusts (FDR < 0.01). Gray dots represent genes with no significant change in TE. The total gene number used for analysis is shown in parentheses. (*B*) Changes in mRNA abundance and TE in gregarious locusts compared with solitary locusts. Log_2_ of the mRNA fold change is shown on the horizontal axis, and log_2_ of the TE fold change is shown on the vertical axis. Differentially expressed genes at the transcriptional and translational levels are shown in parentheses (*Left*). The proportions of different changes from transcription and translation contribute to TE alteration in pie chart (*Right*). (*C*) Pathway analysis of genes with increased TE in gregarious locusts. (*D*) Pathway analysis of genes with increased TE in solitary locusts. (*E*) Differentially expressed genes from the ribosome pathway with increased TE in gregarious and solitary locusts. The corresponding names in the new nomenclature were provided at the first mention of the RP genes.

We compared the TE values of differentially expressed genes between gregarious and solitary locusts. Among the genes with TE changes, 2,927 and 1,978 up-regulated genes were found in gregarious and solitary locusts, respectively (Fig. 3*A*). We further screened a subset of genes based on translational changes alone by the joint analysis of TE and transcriptome data to exclude the alteration of TE caused by transcriptional changes. In the nine-quadrant diagram, 2,685 genes showed differences only in translation between gregarious and solitary locusts (Fig. 3*B*). Eighty percent of TE changes resulted from translational changes alone (Fig. 3*B*). The genes with high TE in gregarious locusts were enriched in oxidative phosphorylation and ribosome pathways (Fig. 3*C*). In contrast, more genes from pyrimidine metabolism and ribosome pathway had increased TEs in solitary locusts (Fig. 3*D*). The common ribosome pathway was enriched in the two phases of locusts, but divergent genes were observed between gregarious and solitary locusts. A total of 10 RP genes among the 13 ribosome pathway genes with higher TE in gregarious locusts were from the cytoplasm, and 5 RP genes among the 6 ribosome pathway genes that were translationally up-regulated in solitary locusts were from the mitochondria (Fig. 3*E*). The gregarious locusts preferentially increased the TE of large RPs (6/10) from the cytoplasm, whereas more of the large RPs (4/5) from mitochondria had a higher TE in solitary locusts (Fig. 3*E*). This is also consistent with the mass spectrometry results for the 60S fractions in polysome profiles (Fig. 1*H*).

**Fig. 4.**
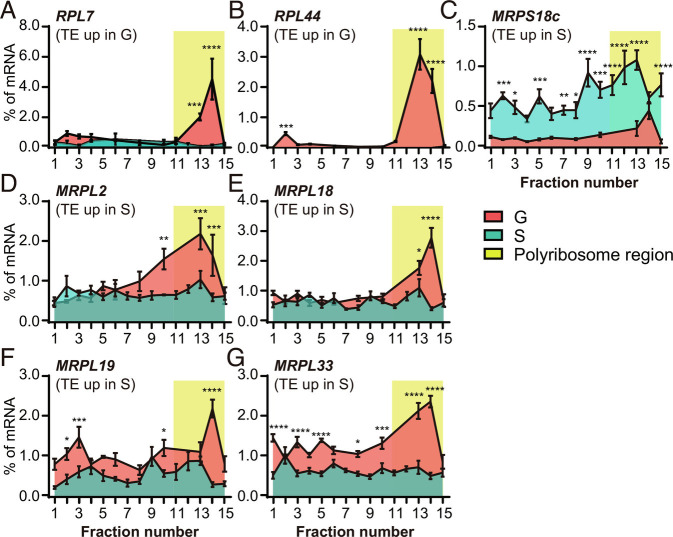
Changes in TE of ribosomal protein genes between gregarious and solitary locusts. (*A* and *B*) The relative mRNA levels of two up-regulated ribosomal protein genes in gregarious locusts were measured by qPCR performed from the top (fraction 1) to the bottom (fraction 15) of the polysome gradient. (*C–G*) The relative mRNA levels of five up-regulated ribosomal protein genes in solitary locusts were measured by qPCR from the top (fraction 1) to the bottom (fraction 15) of the polysome gradient. Expression values were normalized based on the addition of equal amounts of luciferase RNA to each fraction prior to RNA extraction. Standard error of mean were calculated from three biological repeats. *P* values were calculated using a *t* test, with *P < 0.05, **P < 0.01, ***P < 0.001, ****P < 0.0001.

Additionally, we monitored the distribution of mRNAs from RP genes with different TEs in the polysome profiling fractions to further determine the genes involved in translational regulation. Notably, the mRNAs of *RPL7* and *RPL44* from the cytoplasm were highly distributed in the polysome fractions in gregarious locusts (Fig. 4 *A* and *B*), indicating an active translation state. Conversely, we verified that the mRNA of *MRPS18c* from mitochondria shifted into larger polysomes in solitary locusts (Fig. 4*C*). This change in trend is consistent with the changes in the TE of RPs between gregarious and solitary locusts. However, the distribution of *MRPL2*, *MRPL18*, *MRPL19,* and *MRPL33* mRNA is inconsistent with the change in trends in TE (Fig. 4 *D*–*G*). Thus, the gregarious and solitary locusts employed divergent ribosome pathway genes with TE changes.

### RPL7 and MRPS18c-Mediated Polysome Profiles and Behavioral Shift in Locust Phase Change.

**Fig. 5.**
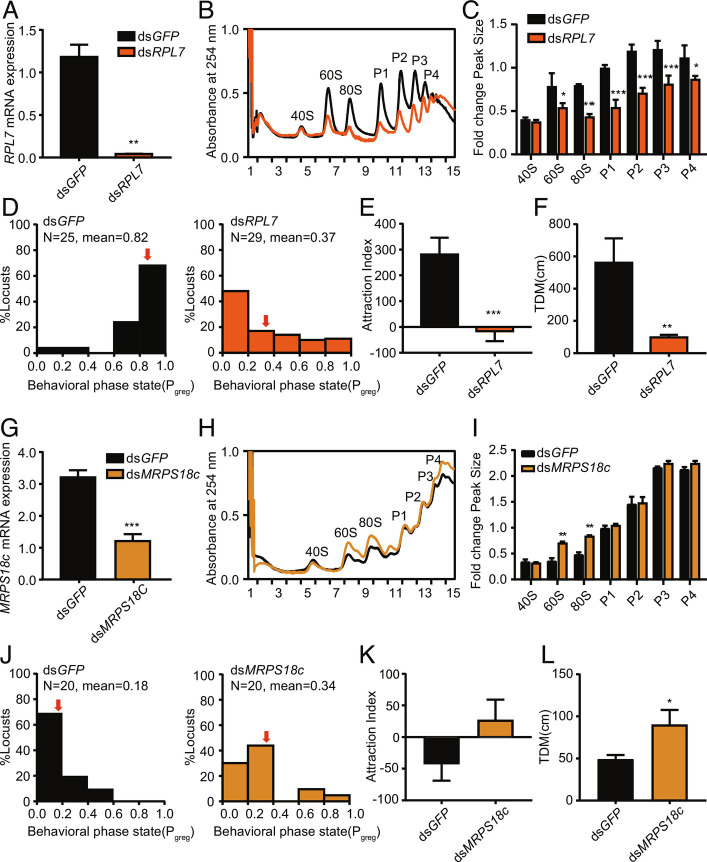
Contributions of *RPL7* and *MRPS18c* genes to the changes in polysome profiles and behavioral shift in locust phase transition. (*A–F*) Effects on RNAi, polysome profile, behavioral phase change (P_greg_), attraction index, and total distance of movement (TDM) caused by injecting dsRNA of *RPL7* into gregarious locusts. (*G–L*) Effects on RNAi, polysome profile, behavioral phase change (P_greg_), attraction index, and total distance of movement (TDM) caused by injecting dsRNA of *MRPS18c* into solitary locusts. Quantification of polysome peak sizes of gregarious and solitary locusts in representative experiments with n = 3, normalized to the P1 peak of ds*GFP* locusts. Dark columns represent the control group (ds*GFP*). Orange columns represent the treated group. Red arrows show the mean values of P_greg_. “N” indicates the number of locusts in each group. Bars represent the mean ± SEM, and significance was tested with Student’s *t* test. *P* values were calculated using a *t* test, with *P < 0.05, **P < 0.01, ***P < 0.001.

We performed RNA interference in locusts to verify the functions of the RP genes with TE changes in phase transition. The RNA interference of *RPL7* and *MRPS18c* led to significant changes in the molecular phenotypes of polysome profiles in gregarious and solitary locusts (Fig. 5 *A*–*C* and *G*–*I*). Compared with the control, the locusts with *RPL7* knockdown exhibited markedly decreased absorbance of 60S, 80S, and P1-P4 polyribosomes, while the locusts with ds*RPL44* showed no significant change (Fig. 5 *A*–*C* and *SI Appendix*, Fig. S6 *A–C*). The behavior assay showed that the P_greg_ values (P_greg_ value = 1 means typical gregarious phase) of *RPL7* and *RPL44* dramatically decreased from 0.82 to 0.37 and from 0.91 to 0.51, respectively (Fig. 5*D* and *SI Appendix*, Fig. S6*D*). Gregarious locusts with knockdown of *RPL7* and *RPL44* shifted toward solitary-like behavior with a reduction in the attraction index (Fig. 5*E* and *SI Appendix*, Fig. S6*E*). Moreover, the *RPL7* and *RPL44* knockdown locusts exhibited a shorter total distance of movement (Fig. 5*F* and *SI Appendix*, Fig. S6*F*). After the injection of ds*MRPS18c* in solitary locusts, the absorbance of 60S and 80S significantly increased and shifted toward that in gregarious-like polysome profiles (Fig. 5 *G*–*I*). Moreover, the P_greg_ value of *MRPS18c* knockdown locusts increased from 0.18 to 0.34 with a positive attraction index, whereas the total distance of movement were increased (Fig. 5 *J*–*L*). Therefore, the expression changes in *RPL7* and *MRPS18c* at the translational level may play an important role in the molecular phenotype and behavioral transition.

The gene *pale*, which is a critical dopamine synthesis gene without differential mRNA expression between solitary and gregarious locusts, can nevertheless lead to behavioral aggregation of locusts through differential protein expression (11). This clue implies that the *pale* gene may be regulated at the translational level. RNA-binding protein immunoprecipitation assays showed the binding of *pale* mRNA to RPL10A, indicating the potential influence of RPL7 on the translation of *pale*. After knockdown of *RPL7* by RNAi, the binding of *pale* mRNA to RPL10A decreased significantly to 50% (*SI Appendix*, Fig. S7 *A* and *B*). Meanwhile, the *pale* mRNA were enriched in the polysome fractions of gregarious locusts, indicating more ribosome loading onto *pale* mRNA in gregarious locusts compared with solitary locusts (*SI Appendix*, Fig. S7*C*). These data indicate that *RPL7* promotes *pale* mRNA loading to ribosomes by impacting translation, which modulates behavioral changes from solitary to gregarious locusts. Furthermore, we inferred that *RPL7* promoted the phase change from the solitary to the gregarious phase because the ribosome abundance could be regulated by *RPL7*.

## Discussion

**Fig. 6.**
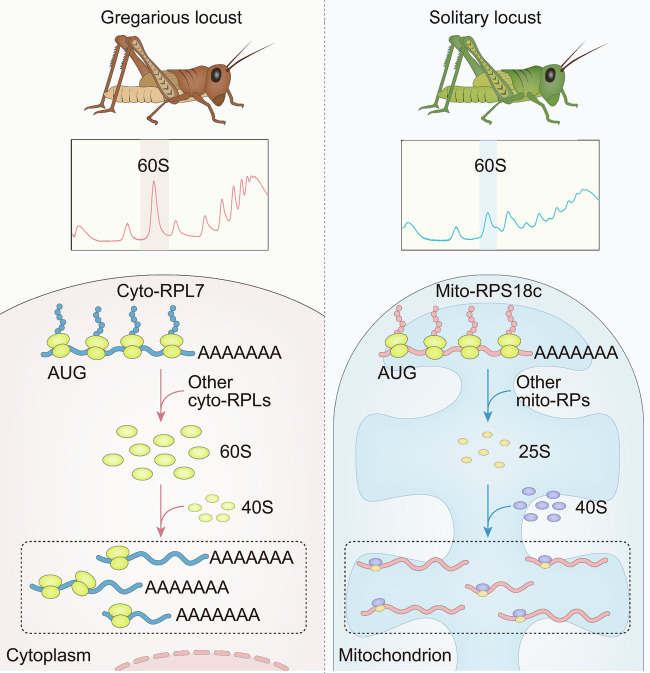
Translational regulation effect of RPs from cytoplasm and mitochondria on the divergence of ribosome profiles in gregarious and solitary locusts. In gregarious locusts, the high expression of cyto-RPL7 the translational level or other cyto-RPLs promotes accumulation of the free 60S ribosomal subunit in cytoplasm (*Left*). By contrast, solitary locusts employ *MRPS18c* or other mito-RPs to induce the assembly of mitochondrial ribosomes, causing divergence of the translational profiles and behavioral transition (*Right*).

The phase change in locusts, as a typical instance of phenotypic plasticity in insects, is regulated by multiple molecular mechanisms, including neurotransmitters, transcription, posttranscription of coding genes, and noncoding RNAs (9, 10, 12, 13, 29). In contrast, our present study uncovers an entirely different regulatory mechanism at the translational level, in which gregarious and solitary locusts employ divergentpolysome profiles in cytosolic and mitochondrial ribosomal proteins (Fig. 6). Higher translation of cyto-RPL7 in gregarious locusts can induce high 60S abundance, while disrupting the expression of MRPS18c in solitary locusts can increase 60S abundance. Therefore, the cytosolic and mitochondrial ribosomes mediate the divergent translational strategy in the locust phase transition.

The molecular phenotypes of translational levels between solitary and gregarious locusts are involved in polysome profiling. Gregarious locusts have more 60S subunits and polyribosomes than solitary locusts. The density of polyribosomes is closely related to the TE (31, 32). Thus, gregarious locusts may have more genes with high TE than solitary locusts. Interestingly, a specific half-mer peak after the 60S, 80S, and polyribosome peaks appeared in the solitary locusts, for which we suspected to be attributable to the slow 80S formation caused by the stalled 40S ribosomal subunit binding mRNA but lacking 60S subunits. The reduction in the amount of 60S subunits relative to 40S subunits blocks their joining step in the ribosome assembly process and leads to translation initiation defects, resulting in similar half-mer peaks in yeast (33–35). We knockdown the RPL7 showed a global reduces in 60S, 80S, and polyribosomes peaks, no half-mer peaks appeared yet. This indicates that other ribosomal components are responsible for half-mer peaks. It will be very necessary to search the factors for halfmers. Thus, solitary locusts with half-mer peaks have fewer latent intact ribosomes than gregarious locusts. The assembly of an intact ribosome including more than 200 proteins and other factors is a concerted molecular process named ribosome biogenesis (RiBi). RiBi is a common aa differentiation “switch” during stem cell differentiation (36). Stem cells from embryos and adults accumulate latent intact ribosomes to rapidly synthesize the proteomes of differentiated cells through the up-regulated translation of RPs (37, 38). The behavioral transition in the time course between solitarization and gregarization exhibits asymmetry in the migratory locust. The behavioral solitarization of fourth-instar gregarious nymphs is considerably faster, whereas the gregarization of solitary nymphs occurs slowly (9). More latent intact ribosomes in gregarious locusts enable the rapid synthesis of a proteome trend in solitary locusts after solitarization. Conversely, solitary locusts have fewer latent intact ribosomes to slowly alter the gregarious-like proteome after gregarization. Therefore, RiBi may act as a time switch for behavioral transition in locusts.

Gregarious and solitary locusts employ divergent ribosome profiles in cytosolic and mitochondrial RPs. Solitary locusts have more active MRPs to enhance energy metabolic gene translation for high-level energy metabolism. Thus, solitary locusts exhibit robust energy metabolism activity, which contributes to their higher distance moved in the first jump in fourth-instar nymphs than that of gregarious locusts (39). It also results in high initial flight speeds and short-term flight of adult solitary locusts (5). Moreover, solitary locusts slow cytosolic mRNA translation, leading to their longer lifespan than that of gregarious locusts. This phenomenon is consistent with the ability of cytosolic translation inhibition to extend lifespan in yeast, worms, flies, human cells, and mice by reducing the expression of RPs (30). Unlike the mitocytosolic translational balance mechanism in worms, human cells, and mice, gregarious and solitary locusts employ divergence of translational profiles and not synergetic balance (30). Mutations in the genes encoding MRPs disrupt translational balance and ultimately cause severe neurological defects, depletion in heart and skeletal muscle, and cardiovascular disorders (40). Therefore, further studies are needed to elucidate the biological function of translation divergence in mitochondria-rich tissues in locusts.

RPL7, as a large ribosomal subunit protein, plays a critical role in RiBi in eukaryotes (41, 42). In this study, RPL7 is shown to play a critical role in forming different translation patterns between solitary and gregarious locusts. These results support the hypothesis that different polysome profiles between gregarious and solitary locusts result in different translation strategies. The high expression of RPL7 at the translational level promotes accumulation of the free 60S ribosomal subunit, contributing to fast polyribosome formation and further enhancement in molecular phenotype transition. RPL7 may play a feedback regulatory role in the locust phase transition. We showed that RPL7, RPL44, and MRPS18c with TE changes responded to population density changes and subsequently led to behavioral transition. Further in-depth analysis and identification of upstream regulatory factors for global translational alteration will be important in understanding the locust response to the population density.

We revealed that translational regulation using ribosome profiling is a fundamental layer of phenotypic plasticity in locusts. Previous research focused primarily on the transcriptional and posttranscriptional regulations, which play important roles in phenotypic plasticity of locust (9, 10, 12, 13, 43). Our present results suggest that the locusts rapidly change the expression of a large number of genes at the translational level to respond to changes in population density. Dynamic translational control in response to light (44), heat (31), cold (45), drought (46), hormone (47), nutrition (27), pathogen (28), and hypoxia (26) stress is important for stress adaptation and survival in plants. In humans, individual genetic differences near start codons and upstream open reading frames specifically modulate ribosome occupancy for individual protein level differences, which is crucial for understanding phenotypic diversity, disease susceptibility, and personalized medicine (48). In brief, an evolutionarily conserved strategy of translational control is widely used by organisms to respond to internal and external environmental variations rapidly and efficiently.

The locust phase transition exhibits a striking phenotypic plasticity of behavior, in which dopamine and its synthesis enzyme genes, including *pale* and *henna* in this pathway, play important regulatory roles through the transcriptional regulation of this process (11, 14, 43). We further observed that the TE of the *RPL7* and *RPL44* transcripts in gregarious locusts was higher, facilitating behavioral changes from the solitary to the gregarious phase. Moreover, defects in *RPL7* cause a decline in ribosome binding with *pale*. In previous research, *pale* mRNA showed no significant differential expression between the two phases of locusts, but higher expression at the protein level was noted in gregarious locusts than in solitary locusts (14). Our results suggest that the phase changes in locusts induce *pale* protein by increasing *RPL7*-promoted *pale* mRNA on ribosomes for translation. Decreases in *RPL44* also inhibit the attraction of gregarious locusts, but *RPL44* has no influence on *pale* mRNA loading onto ribosomes. *RPL44* in locusts might regulate behavior through other pathways. Beyond canonical ribosomal assembly functions, human RPs interact with non-RPs to control cell growth, proliferation, differentiation, immune signaling, DNA repair, and apoptosis by extraribosomal functions (49). RP mutations are highly associated with human diseases such as cancer, which is why a diverse range of anticancer drugs target ribosomes (49). Thus, two RPs obtained by translational regulation can be used to prevent aggregation to control locust outbreaks. Our data showed that the effects of *RPL7* or *RPL44* knockdown on behavioral transition were direct and indirect, respectively. The *RPL7* knockdown locusts had a global reduction in 60S, 80S, and polyribosomes peaks, and could affect bind to *pale* mRNA for behavioral transition. This indicates that RPL7 is a core component of ribosome. However, there was no change of ribosome profiles and affect bind to *pale* mRNA in *RPL44* knockdown locusts. We speculated that RPL44 may recruit other mRNAs to the ribosome or has extraribosomal functions for behavioral transition. In mouse embryonic stem cells, the translating ribosomes are heterogeneous in core RPs (50). A subset of mRNAs genome-wide is preferentially translated by heterogeneous ribosomes. For example, RPL10A/uL1 preferentially translates a subset of mRNAs with IRES (internal ribosome entry site) elements in 5′ UTR (50). RPs as a ribosomal component have a specialized function in translation of particular mRNA, regarded as ribosome heterogeneity (51). Uncovering how the heterogeneous ribosomes preferentially translate particular genetic networks at the whole genomic level in insects is challenging.

## Materials and Methods

### Insect Materials.

The locusts used in this study were obtained from the Institute of Zoology, CAS, China, and maintained for more than 20 generations. Gregarious locusts were reared in metal cages with a size of 25 cm × 25 cm × 25 cm at population densities of 300 to 400 insects per cage. The solitary locusts were individually reared in separate cages with circulating air through carbon filters. All of the locusts were grown to the fourth-instar under a stable photoperiod (light:dark = 14 h:10 h) and temperature (30 °C ± 2 °C).

### Polysome Profiling.

The locust samples were ground into powder with liquid nitrogen. A polysome extraction buffer (0.2 M Tris-HCl [pH 9.0], 0.2 M KCl, 25 mM EGTA, 35 mM MgCl_2_, 1% [w/v] Brij-35, 1% [v/v] Triton X-100, 1% [v/v] Igepal CA630, 1% [v/v] Tween 20, 1% [w/v] DOC, 1% [v/v] polyoxyethylene [10] tridecyl ether, 5 mM DTT, 1 mM PMSF, 50 μg/mL cycloheximide and chloramphenicol) was added to the powder to extract the total ribosomes of locusts.

The ribosomes were pelleted by ultracentrifugation at 4 °C overnight through a sucrose cushion (0.4 M Tris-HCl [pH 9.0], 0.2 M KCl, 5 mM EGTA, 35 mM MgCl_2_, 1.75 M sucrose, 5 mM DTT, 50 μg/mL cycloheximide and chloramphenicol). The ribosome pellet was dissolved in RNase I digestion buffer (20 mM Tris-HCl [pH 8.0], 0.14 M KCl, 5 mM MgCl_2_, 50 μg/mL cycloheximide and chloramphenicol) and separated by ultracentrifugation with a 5 to 50% sucrose gradient at 4 °C and 35,300 rpm (SW41 rotor, Beckman) for 3 h. Profiling was performed using a piston gradient fractionator (Biocomp), and signals were detected at 254-nm UV absorbance.

### Ribo-seq.

Each 5,000 U total ribosomes were digested by 1,500 U RNase I (Ambion, AM2294) for RF generation. The digested solutions were separated by profiling as described in the polysome profiling experiments. We added 2 volume 8 M guanidine hydrochloride, 3 volume ethanol, and 2 µL GlycoBlue (Ambion, AM9516, 15 mg/mL) into monosome fractions for RNA extraction. The ~28-nt RFs were separated with Urea-PAGE, and rRNA was removed using DNA probes complementary to rRNA sequences. Then, RNase H and DNase I were used to digest the probes. RFs were purified using magnet beads (Vazyme). After obtaining RFs above, Ribo-seq libraries were constructed using NEBNext Multiple Small RNA Library Prep Set for Illumina (catalog no.E7300S, E7300L). Briefly, adapters were added to both ends of RFs, followed by reverse transcription and PCR amplification. The 140 to 160-bp size PCR products were enriched to generate a cDNA library and sequenced using Illumina HiSeqTM 2500 by Gene Denovo Biotechnology Co.

### RNAi.

Double-stranded RNAs were synthesized with the T7 RiboMAX™ Express RNAi System (Promega, P1700) according to the instructions in the kit manual. The primers used for RNAi are listed in the supplemental materials *SI Appendix*, Table S3. The dsRNA solutions were diluted into 0.625, 1.25, 2.5, and 5 μg/μL concentrations to select the effective interference. In our study, the 0.625 μg/μL was chosen for candidate genes RNAi. A fixed-volume (2 μL) dsRNA solution was injected into hemolymph of fourth-instar locusts at the second abdominal segment toward the head. The control group locusts were injected dsRNA of *GFP* (ds*GFP*) with the same concentration, volume, and the stage of injection. The mRNA expression detection of target genes and behavioral test was performed 72 h after injection.

### Behavioral Test.

As previous study described (9), P_greg_ = 1 defined typical gregarious behavior, and P_greg_ = 0 defined typical solitary behavior. The locusts were tested one by one in a rectangular Perspex arena (40 cm long × 30 cm wide × 10 cm high) using an EthoVision video tracking system (Noldus Information Technology). Two separated chambers were set on two sides of the arena, one of them contained 15 fourth-instar gregarious locusts for stimulating and the other kept empty. The locusts were released into the arena and moved freely 2 min for adapting to the environment; then, the tracks of locusts were monitored for 5 min. The data were recorded automatically. The behavioral phase state of each individual was calculated with a binary logistic model, P_greg_ = e^η^/(1 + e^η^), η = −2.110 + 0.005 × attraction index + 0.012 × total distance moved + 0.015 × total duration of movement. In this model, attraction index means the sum of weighted total duration in three areas of the arena, which is equal to 1× time in stimulus area + 0× time in middle area + (−1) × time in the opposite of stimulus area. Total distance moved and total duration of movement were provided by EthoVision video tracking system software.

Details of other methods are in *SI Appendix*, *Methods*.

## Supplementary Material

Appendix 01 (PDF)

## Data Availability

The sequencing data were deposited in the Genome Sequence Archive Database (accession number: PRJCA014381).The mRNA-seq  data were CRA009541 and Ribo-seq data were CRA009542. All study data are included in the article and/or *SI Appendix*.
