# Supplementary material for: Cytosolic and mitochondrial ribosomal proteins mediate the locust phase transition via divergence of translational profiles
Source: Proc Natl Acad Sci U S A. 2023 Jan 26;120(5):e2216851120. doi: 10.1073/pnas.2216851120 (PMC9945961; doi:10.1073/pnas.2216851120)
Supplement: Supplementary file 1 — Appendix 01 (PDF) [file pnas.2216851120.sapp.pdf]

PNAS

Supporting Information for

Cytosolic and mitochondrial ribosomal proteins mediate the locust phase transition via divergence of translational profiles

Jing Li [a,1], Liya Wei[a,1,2], Yongsheng Wang[a], Haikang Zhang[a], Pengcheng Yang[b], Zhangwu Zhao[a], and Le Kang[a,b,c,2]

[a] College of Life Science, Institute of Life Science and Green Development, Hebei University, Baoding 071002, China

[b] Beijing Institutes of Life Science, Chinese Academy of Sciences, Beijing 100101, China

[c] Institute of Zoology, State Key Laboratory of Integrated Management of Pest Insects and Rodents, Chinese Academy of Sciences, Beijing 100101, China

[1]J.L. and L.W. contributed equally to this work.

[2]To whom correspondence may be addressed. Email: weiliya@hbu.edu.cn or lkang@ioz.ac.cn.

This PDF file includes:

Supporting text
Figures S1 to S7
Tables S1 to S3

Supporting Information Text

Methods

mRNA-seq

Total RNA was extracted using a TRIzol reagent kit (Invitrogen, USA). RNA quality was assessed on an Agilent 2100 Bioanalyzer (Agilent Technologies, Palo Alto, CA, USA) and checked by RNase-free agarose gel electrophoresis. mRNA was enriched from total RNA with Oligo (dT) beads, and rRNA was removed by a Ribo-Zero™ Magnetic Kit (Epicenter, Madison, USA). The mRNA was fragmented into short segments and reverse transcribed into cDNA with random primers. Then, the cDNA fragments were purified by a QiaQuick PCR extraction kit (Qiagen, The Netherlands). End repair, poly (A) addition, and Illumina sequencing adapter ligation were performed for sequencing. The ligation products were selected on the basis of size using agarose gel and sequenced through Illumina HiSeq2500 by Gene Denovo Biotechnology Co. (Guangzhou, China).

RNA-binding protein immunoprecipitation assay

A RIP Kit (BersinBio, Bes5101) was used in this experiment. Total protein was extracted by TRIzol and separated into three parts for input, IgG, and RPL10A incubation. According to the manuals, 5 μg of RPL10A antibody was used in each reaction. After immunoprecipitation, the mRNAs in each reaction were collected by RNA extraction solution (phenol:chloroform:isoamyl alcohol=25:24:1, pH<5) and pelleted with ethanol. The RNA was dissolved in nuclease-free water and reverse transcribed by M-MLV Reverse Transcriptase (Promega, M1705). *q*PCR was performed with SsoFast EvaGreen Supermix (BIORAD, 1725202). The primers for *q*PCR are listed in the supplemental materials Table S3.

Bioinformatics analysis for mRNA-seq and Ribo-seq

We initially removed low-quality reads with the default parameters of the HTQC package (v-1.92.1) to compare gene expression between gregarious and solitary locusts (1), and then the remaining reads were mapped to the Locust genome (2) by HISAT2 (v-2.2.1) (3). The expression of genes was normalized by fragments per kilobase million based on unique mapping reads, and clustering analysis was performed using R. Differentially and transcriptionally expressed genes were detected with fold change ≥ 1.4 and *P value* < 0.05 as cutoffs, in which the *P* value and FDR were calculated using the DEGseq package (4-6).

The remaining Ribo-seq reads were aligned to the reference genome (2) using HISAT2 (v-2.2.1) (3) after removing the rRNA, tRNA, snRNA, and snoRNA. The unique mapping reads were used to calculate the length distribution of reads protected by ribosomes; the frequency distribution in the intergenic region of the genome; and the 5′ UTR, CDS, 3′ UTR, and intron region of the annotated gene. The unbiased metagene profiles were generated by counting 18–40-nt unique mapping reads at each position. Each read was represented by the first nucleotide mapping in the genome and normalized per million mapped reads (RPM). The TE was calculated

by RPM in the CDS of Ribo-seq divided by RPM in the CDS of mRNA-seq (5, 6). The significantly and differentially expressed genes at the translational level were defined using the same parameter by mRNA-seq. Clustering analysis of translational datasets was performed on TE.

We used the nine-quadrant diagram to identify genome-wide transcriptional and translational differences in gregarious and solitary locusts. There were five expression patterns, such as transcriptional changes alone, translational changes alone, opposite changes, homodirectional changes, and no changes. The opposite changes represent one gene with opposite changes at transcriptional and translational levels. The homodirectional changes represent one gene with consistent changes at transcriptional and translational levels.

UTRs annotated based on third-generation sequencing

Previous PacBio and Nanopore read (7) datasets were corrected and mapped to the locust genome by Minimap2 (8) using default parameters. The longest unique mapping reads of each gene were retained to annotate the UTRs using the following parameter: reads exceeded the start and end positions of the gene by at least 20 bp.

Fig. S1.

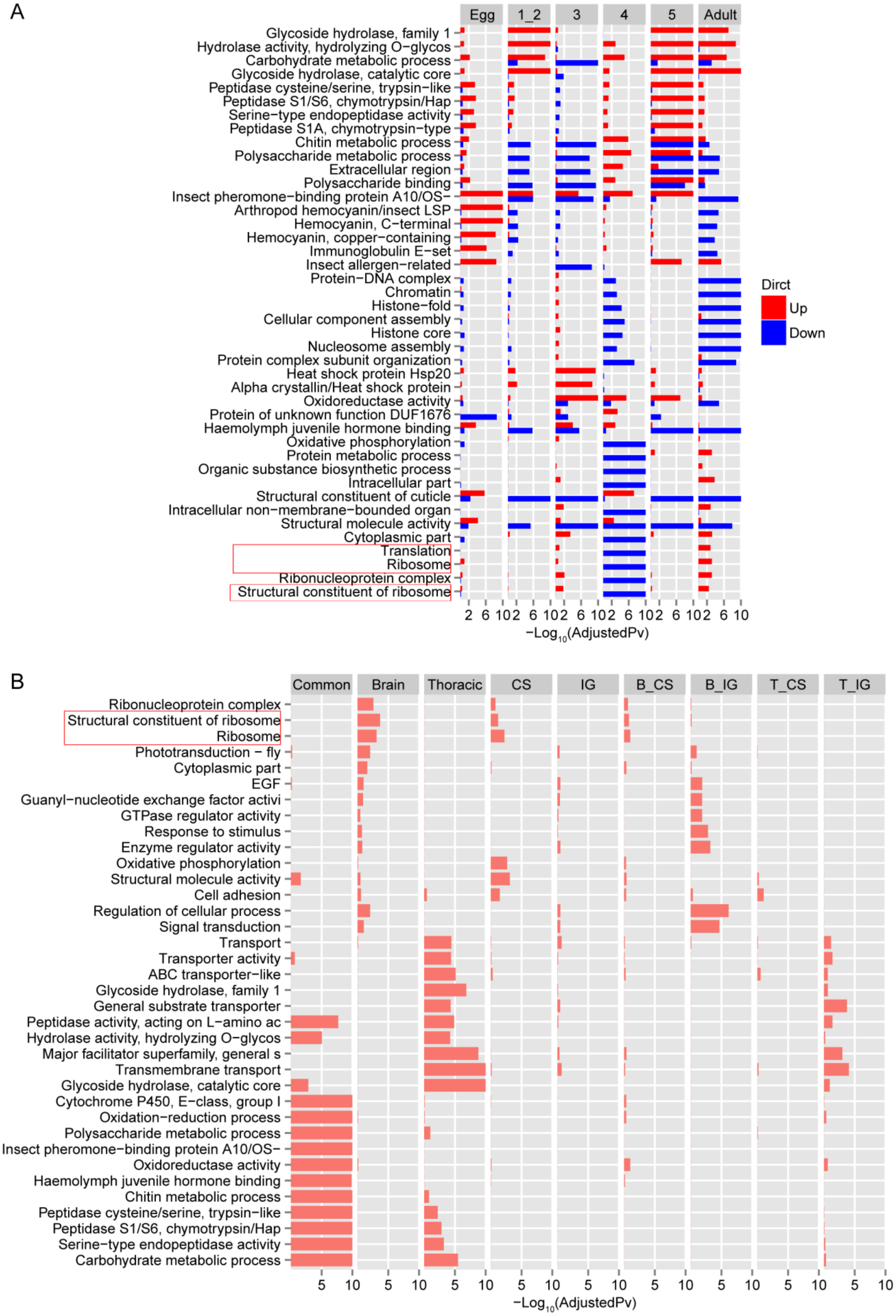
A
Egg
1_2
3
4
5
Adult
Glycoside hydrolase, family 1
Hydrolase activity, hydrolyzing O−glycos
Carbohydrate metabolic process
Glycoside hydrolase, catalytic core
Peptidase cysteine/serine, trypsin−like
Peptidase S1/S6, chymotrypsin/Hap
Serine−type endopeptidase activity
Peptidase S1A, chymotrypsin−type
Chitin metabolic process
Polysaccharide metabolic process
Extracellular region
Polysaccharide binding
Insect pheromone−binding protein A10/OS−
Arthropod hemocyanin/insect LSP
Hemocyanin, C−terminal
Hemocyanin, copper−containing
Immunoglobulin E−set
Insect allergen−related
Protein−DNA complex
Chromatin
Histone−fold
Cellular component assembly
Histone core
Nucleosome assembly
Protein complex subunit organization
Heat shock protein Hsp20
Alpha crystallin/Heat shock protein
Oxidoreductase activity
Protein of unknown function DUF1676
Haemolymph juvenile hormone binding
Oxidative phosphorylation
Protein metabolic process
Organic substance biosynthetic process
Intracellular part
Structural constituent of cuticle
Intracellular non−membrane−bounded organ
Structural molecule activity
Cytoplasmic part
Translation
Ribosome
Ribonucleoprotein complex
Structural constituent of ribosome
Dirct
Up
Down
−Log10(AdjustedPv)
B
Common
Brain
Thoracic
CS
IG
B_CS
B_IG
T_CS
T_IG
Ribonucleoprotein complex
Structural constituent of ribosome
Ribosome
Phototransduction − fly
Cytoplasmic part
EGF
Guanyl−nucleotide exchange factor activi
GTPase regulator activity
Response to stimulus
Enzyme regulator activity
Oxidative phosphorylation
Structural molecule activity
Cell adhesion
Regulation of cellular process
Signal transduction
Transport
Transporter activity
ABC transporter−like
Glycoside hydrolase, family 1
General substrate transporter
Peptidase activity, acting on L−amino ac
Hydrolase activity, hydrolyzing O−glycos
Major facilitator superfamily, general s
Transmembrane transport
Glycoside hydrolase, catalytic core
Cytochrome P450, E−class, group I
Oxidation−reduction process
Polysaccharide metabolic process
Insect pheromone−binding protein A10/OS−
Oxidoreductase activity
Haemolymph juvenile hormone binding
Chitin metabolic process
Peptidase cysteine/serine, trypsin−like
Peptidase S1/S6, chymotrypsin/Hap
Serine−type endopeptidase activity
Carbohydrate metabolic process
−Log10(AdjustedPv)

Fig. S1. *(A)* Gene ontology enrichment of differentially expressed genes (DEGs) between solitary and gregarious locusts were analyzed in six different developmental stages from egg to adults, including egg, first to fifth instars, and adults. Red bar denotes the gene ontology enriched for the genes upregulated in gregarious locust, while blue bar denotes those downregulated in gregarious locust. X axis denotes the minus log-transformed *P* values (base 10) of the adjusted p value derived from the enrichment analysis (Fisher's exact test or χ2-test). *(B)* Gene ontology enrichment of DEGs after solitarization (isolating gregarious locusts (IG)) and gregarization (crowding solitarious locusts (CS)) compared with controls. The IG and CS processes in both tissues brain (B) and thoracic (T) tissues were denoted as CS, IG, B_CS, B_IG, T_CS, and T_IG, respectively. The DEGs common to all the B_CS, B_IG, T_CS, and T_IG were denoted as "Common". The controls were denoted as "Brain and Thoratcic". X axis denotes the minus log-transformed *P* values (base 10) of the adjusted *P* value derived from the enrichment analysis (Fisher's exact test or χ2-test).

Fig. S2.

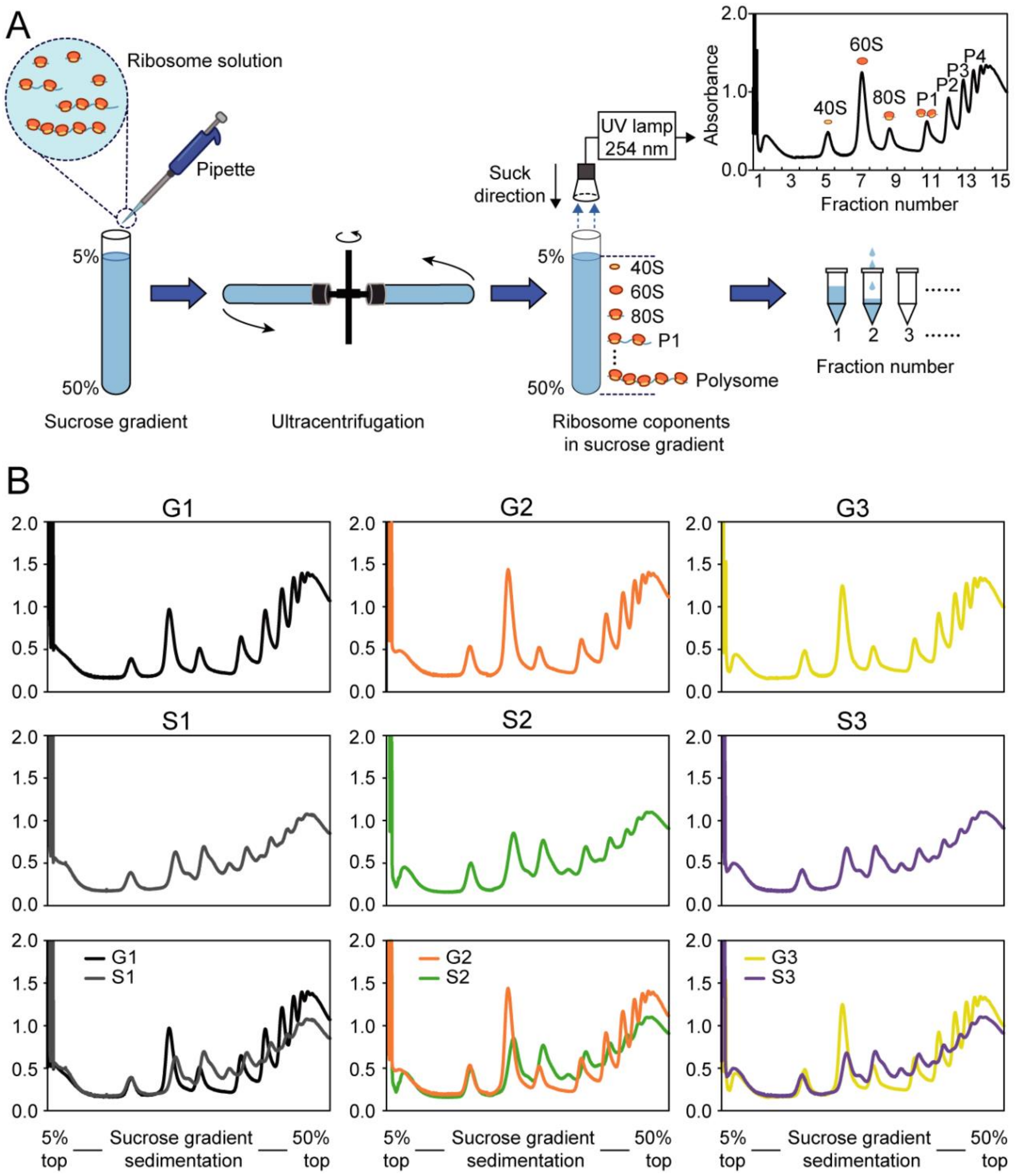

Fig. S2. (*A*) Schematic of the polysome profiling experiment for detecting ribosomal components, including 40S, 60S, 80S, and polyribosomes. (*B*) Polysome profiling analyzed by sucrose gradient sedimentation from three biological replicates of gregarious and solitary locusts represented by G1-3 and S1-3. 10♀10♂ in each group and 5000 U loading quantities for polysome profiling.

Fig. S3.

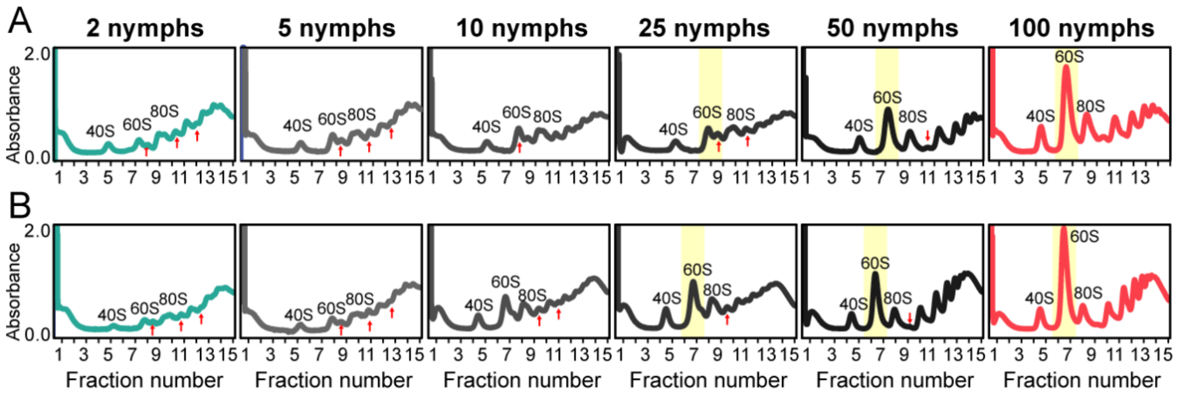

Fig. S3. (*A–B*) Absorbance (A254 nm) of sucrose density gradient fractions measured from ribosomes in locusts at six different population densities, namely, 2, 5, 10, 25, 50, and 100 nymphs from two independent biological replicates beyond Fig. 2. The X-axis indicates the top (fraction 1) to the bottom (fraction 15) from 0 mm to 75 mm of the 5%–50% sucrose gradient. The Y-axis indicates the absorbance (A254 nm) of ribosomes. The red arrowheads indicate peaks specific to solitary locusts. Yellow regions highlight 60S ribosomal subunits.

Fig. S4.

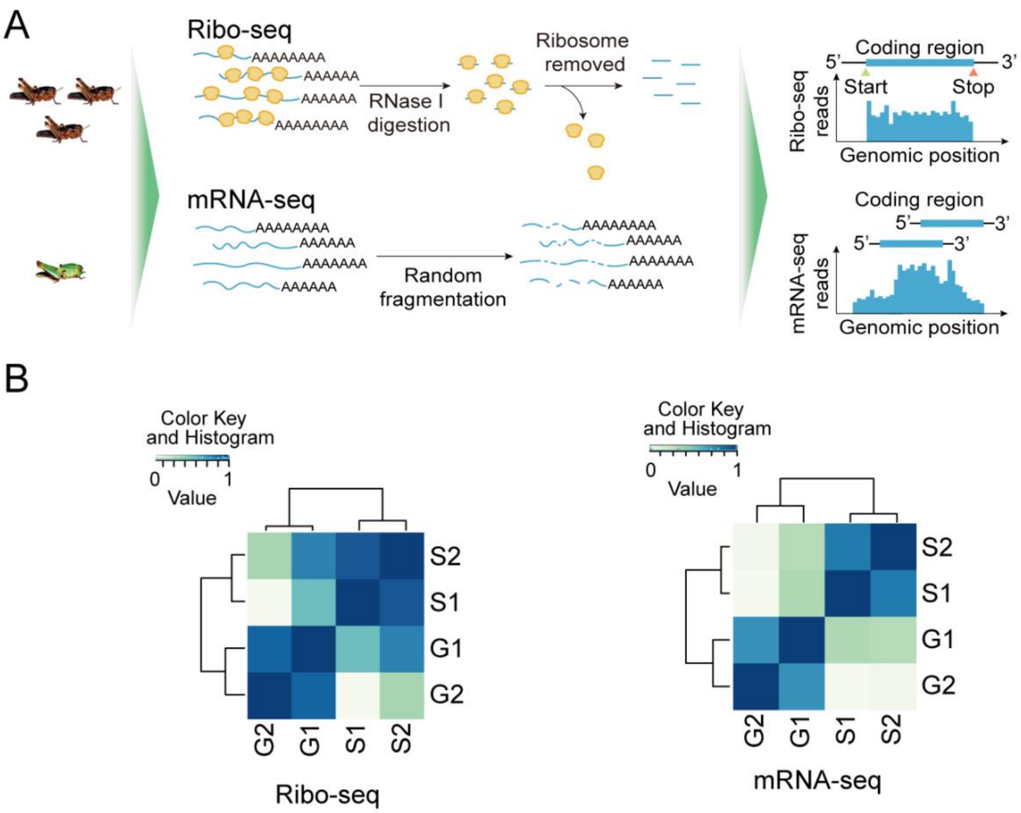

Fig. S4. High-throughput sequencing data indicated gene regulation at the translational level. (*A*) Schematic of the Ribo-seq experiment that sequenced ribosome-protected fragments generated by RNase I (top). Schematic of the mRNA-seq experiment that sequenced mRNAs with poly(A) tails (bottom). (*B*) Reproducibility of the Ribo-seq and mRNA-seq libraries. These high-throughput sequencing samples were extracted from two biological replicates of gregarious and solitary locusts.

Fig. S5.

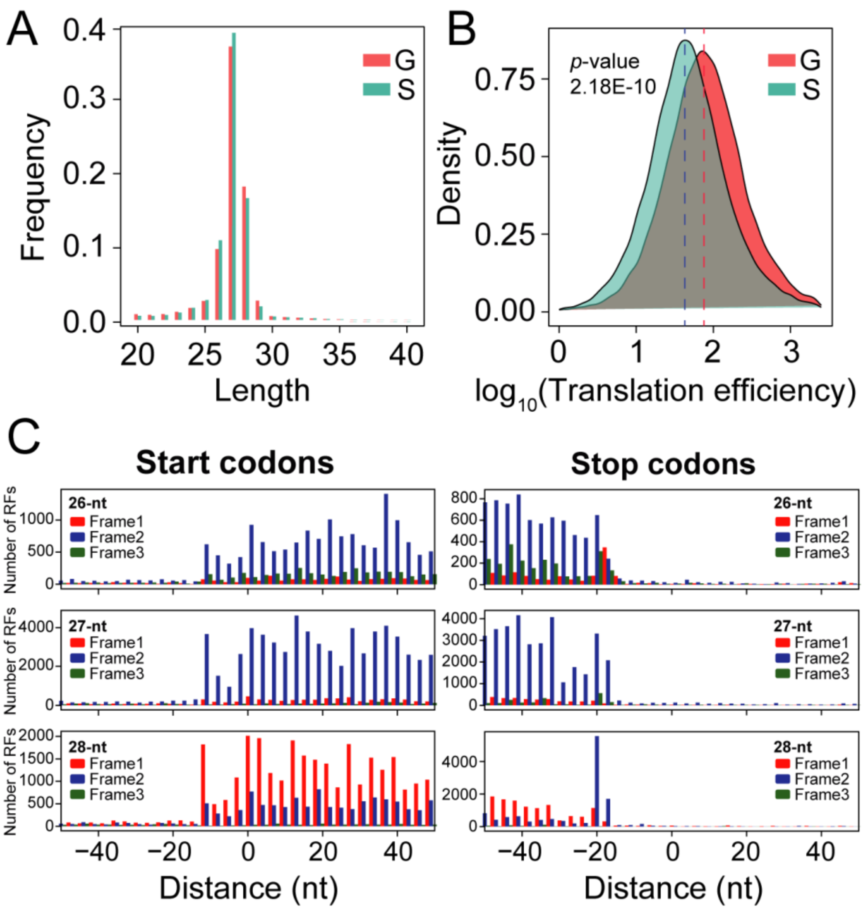

Fig. S5. Length and distribution of ribosomal footprints (RFs) in locusts. (*A*) Size distribution of RFs in gregarious and solitary locusts. (*B*) Density of RFs plotted against TE in gregarious and solitary locusts. We calculated the statistical significance between the distribution of TE between solidary and gregarious locusts by *t* test and obtained that the *P* value is 2.18E-10. (*C*) Bar plot displaying the P-site positions forming 26-nt, 27-nt, and 28-nt RFs across ±50 nt from the start and stop codons of annotated ORFs. The frame position (1, 2, or 3) relative to the transcript is colored red, blue, and green. The P-site of the ribosome is in RFs position 13.

Fig. S6.

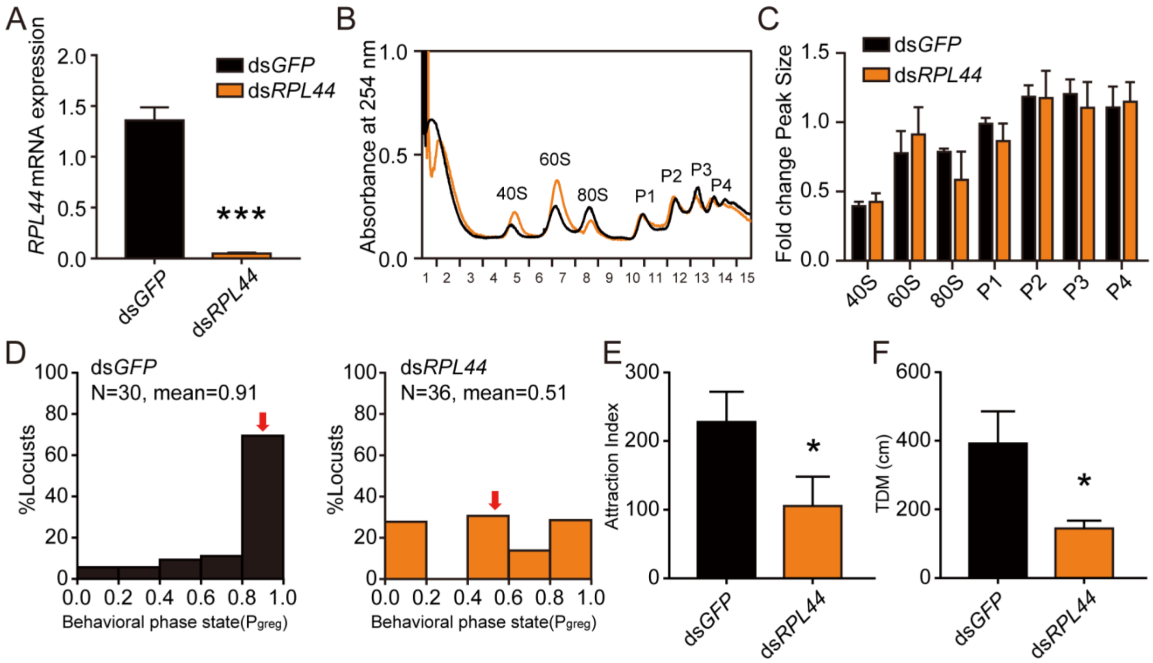

Fig. S6. Effects on RNAi (*A*), polysome profile (*B*-*C*), behavioral phase change (P_{greg}) (*D*), attraction index (*E*), and total distance of movement (*F*) caused by injecting dsRNA of *RPL44* into gregarious locusts. Quantification of polysome peak sizes of locusts in representative experiments with n = 3, normalized to the P1 peak of ds*GFP* locusts. Dark columns represent the control group (ds*GFP*). Orange columns represent the treated group. Red arrows show the mean values of P_{greg}. "N" indicates the number of locusts in each group. Bars represent the mean ± SD, and significance was tested with Student's t test, with *P<0.05, **P<0.01, ***P<0.001.

Fig. S7.

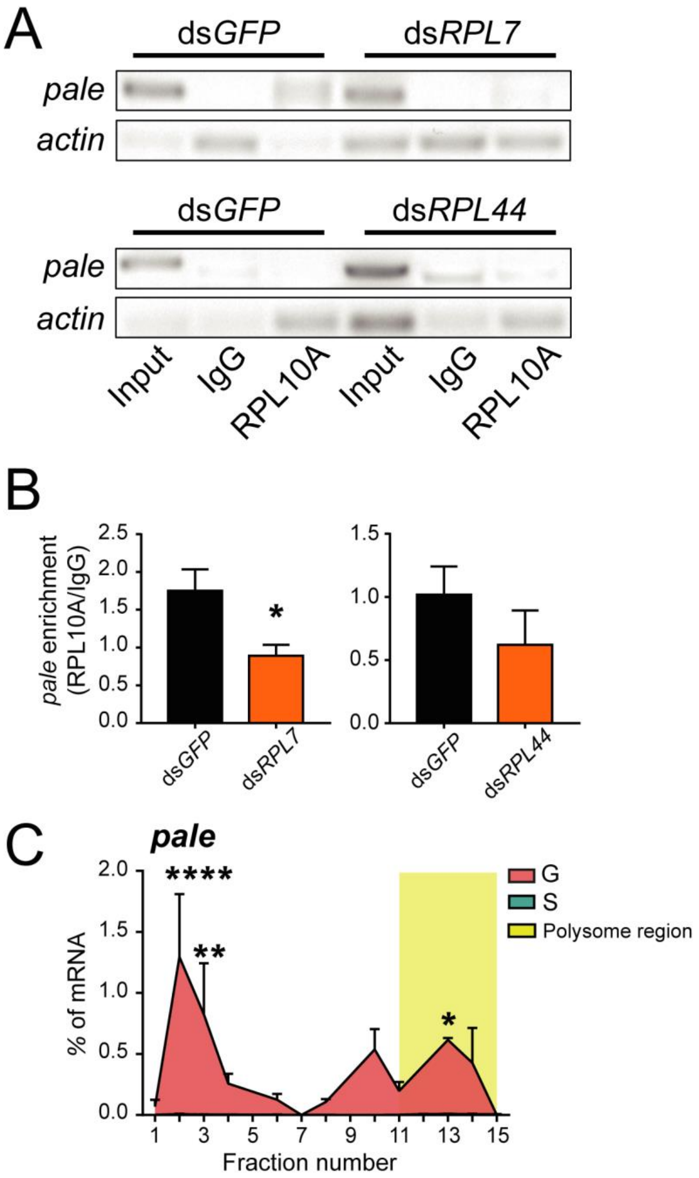

Fig. S7. Enhancement of *pale* mRNA loading to ribosomes by *RPL7*. (*A*) RNA-binding protein immunoprecipitation assays with ds*GFP* (control group) and ds*RPL7* and ds*RPL44* (treated group). *Actin* is the reference gene. IP was performed against RPL10A. (*B*) The enrichment levels of *pale* mRNA in RNA-binding protein immunoprecipitation assays were quantified by Image J software. Each group had four replicates. The data are shown as the mean ± SEM, *P<0.05 (*t* test). (*C*) The relative mRNA levels of *pale* gene were measured by qPCR performed from the top (fraction 1) to the bottom (fraction 15) of the polysome gradient in gregarious and solitary locust. *P* values were calculated using a *t* test, with *P<0.05, **P<0.01, ***P<0.001, ****P<0.0001.

Table S1. Top 10 up-regulated pathways in whole bodies of G adult locusts

Map Title	MapID	Pathway DEGs	*P* value
Glycan structure degradation	Map01032	24	4.78E-15
Glycosaminoglycan degradation	Map00531	15	2.23E-06
Other glycan degradation	Map00511	12	7.51E-05
Oxidative phosphorylation	Map00190	25	1.96E-04
Galactose metabolism	Map00052	16	2.10E-04
Valine, leucine, and isoleucine degradation	Map00280	12	5.35E-04
Folate biosynthesis	Map00790	18	5.97E-04
Propanoate metabolism	Map00640	10	6.56E-04
**Ribosome**	**Map03010**	**29**	**8.19E-04**
RNA polymerase	Map03020	12	9.95E-04

Table S2. Top 10 up-regulated pathways in the fat body of G adult locusts

Map Title	MapID	Pathway DEGs	*P* value
Alanine, aspartate, and glutamate metabolism	Map00250	4	2.94E-03
Hypertrophic cardiomyopathy (HCM)	Map05410	3	3.10E-03
Phosphonate and phosphinate metabolism	Map00511	2	5.32E-03
**Ribosome**	**Map03010**	**7**	**7.33E-03**
Dilated cardiomyopathy	Map05414	3	9.48E-03
Butanoate metabolism	Map00650	3	1.43E-02
Purine metabolism	Map00230	7	1.95E-02
Arrhythmogenic right ventricular cardiomyopathy (ARVC)	Map05412	2	2.67E-02
Other glycan degradation	Map00511	2	3.67E-02
Two-component system	Map02020	2	3.67E-02

Table S3. Primers used in this study

Primer name	Sequence, 5'-3'	Description
RPL7-*T7F*	TAATACGACTCACTATAGGAAACTGAGAAGCCAGCTGCA	RPL7-dsRNA
RPL7-*T7R*	TAATACGACTCACTATAGGTCCCACGGTTTCTTGCTTGC	
RPL44-*T7F*	TAATACGACTCACTATAGGATGGTTAACGTTCCGAAACA	RPL44-dsRNA
RPL44-*T7R*	TAATACGACTCACTATAGGCGTTTCAAAGGAACTTGTTT	
MRPS18c-*T7F*	TAATACGACTCACTATAGGAGGCTATTGGCTTGATGCTC	MRPS18c-dsRNA
MRPS18c-*T7R*	TAATACGACTCACTATAGGGCCATAACACGTCCCAGATT	
GFP-*T7F*	TAATACGACTCACTATAGGGTTCACCTTGATGCCGTTC	GFP-dsRNA
GFP-*T7R*	TAATACGACTCACTATAGGCACAAGTTCAGCGTGTCCG	
RPL7-*rtF*	AAACTGAGAAGCCAGCTGCA	*q*PCR
RPL7-*rtR*	TCCCACGGTTTCTTGCTTGC	
RPL44-*rtF*	ATGGTTAACGTTCCGAAACA	
RPL44-*rtR*	CGTTTCAAAGGAACTTGTTT	
MRPS18c-*rtF*	TGAAGTGATAGCTGCAAGGC	
MRPS18c-*rtR*	ACTTTCGCTTGCTGTCTTGA	
MRPL2-*rtF*	AATCCTTTGGAAAACGTGCC	
MRPL2-*rtR*	TTCGAAATGGACTATCCGGC	
MRPL18-*rtF*	AGGCTATTGGCTTGATGCTC	
MRPL18-*rtR*	GCCATAACACGTCCCAGATT	
MRPL19-*rtF*	CTGAGGGCATCGTTCATTCT	
MRPL19-*rtR*	ACCTTAAGTGGATTCACTGGC	
MRPL33-*rtF*	AAGAAAGCCAAGAGCAAGCA	
MRPL33-*rtR*	TTTGTCCCCTAAGCGTTCTC	
pale-*rtF*	AATCCTGCTTACGTGCTGCT	
pale-*rtR*	TGTAGCATTTAGGCGGAGGT	
GFP-*rtF*	GTTCACCTTGATGCCGTTC	
GFP-*rtR*	CACAAGTTCAGCGTGTCCG	
LUC-*rtF*	CCAGGGATTTCAGTCGATGT	
LUC-*rtR*	AATCTGACGCAGGCAGTTCT	
β-actin-*rtF*	AATTACCATTGGTAACGAGCGATT	
β-actin-*rtR*	TGCTTCCATACCCAGGAATGA	

SI References

1. X. Yang *et al.*, HTQC: a fast quality control toolkit for Illumina sequencing data. *BMC Bioinformatics* **14**, 33 (2013).
2. X. H. Wang *et al.*, The locust genome provides insight into swarm formation and long-distance flight. *Nat. Commun.* **5**, 2957 (2014).
3. D. Kim, B. Langmead, S. L. Salzberg, HISAT: a fast spliced aligner with low memory requirements. *Nat. Methods.* **12**, 357-360 (2015).
4. L. K. Wang, Z. X. Feng, X. Wang, X. W. Wang, X. G. Zhang, DEGseq: an R package for identifying differentially expressed genes from RNA-seq data. *Bioinformatics* **26**, 136-138 (2009).
5. A. Sendoel *et al.*, Translation from unconventional 5′ start sites drives tumour initiation. *Nature* **541**, 494-499 (2017).
6. P. Sharma, J. Wu, B. S. Nilges, S. A. Leidel, Humans and other commonly used model organisms are resistant to cycloheximide-mediated biases in ribosome profiling experiments. *Nat. Commun.***12**, 5094 (2021).
7. F. Jiang *et al.*, Long-read direct RNA sequencing by 5'-Cap capturing reveals the impact of Piwi on the widespread exonization of transposable elements in locusts. *RNA Biology* **16**, 950-959 (2019).
8. H. Li, Minimap2: pairwise alignment for nucleotide sequences. *Bioinformatics* **34**, 3094-3100 (2018).
